# The potential roles of interleukin-25 in infectious diseases

**DOI:** 10.3389/fimmu.2022.986118

**Published:** 2022-09-02

**Authors:** Jing Wu, Fang Zhang, Hongji Tao, Waqas Nawaz, Deyan Chen, Zhiwei Wu

**Affiliations:** ^1^Center for Public Health Research, Medical School of Nanjing University, Nanjing, China; ^2^Department of Burn and Plastic Surgery, Affiliated Hospital of Zunyi Medical University, Zunyi, China; ^3^Hôpital Maisonneuve-Rosemont, School of Medicine, University of Montreal, Montréal, Canada; ^4^State Key Laboratory of Analytical Chemistry for Life Science, Nanjing University, Nanjing, China; ^5^Jiangsu Key Laboratory of Molecular Medicine, Medical School, Nanjing University, Nanjing, China

**Keywords:** IL-17 family, IL-25, Th2, inflammation, infectious diseases

## Abstract

Interleukin-25 (IL-25), also known as IL-17E, is a recently identified cytokine of the IL-17 family. Numerous studies illustrated that the expression of IL-25 is regulated by multiple pathogens, including parasitic, viral, and bacterial infections. IL-25 has a dual function in infectious diseases. On the one hand, IL-25 activates type 2 immunity *via* the relevant cytokines, including IL-4, IL-5, and IL-13, which are associated with the development of pathogenic infection-related allergic diseases. On the other hand, IL-25 involves in the recruitment of group 2 innate lymphoid cells (ILC2) to enhanced T helper 2 (Th2) cell differentiation, which are important to the clearance of pathogens. However, the precise roles of IL-25 in infectious diseases remain largely unknown. Thus, the current review will shed light on the pivotal roles of IL-25 in infectious diseases.

## Introduction

Interleukin-17 (IL-17) was discovered and described in 1993 (1). IL-17 family consists of six members including IL-17A-F (2). IL-17E was also named interleukin-25 (IL-25), which was initially identified by sequence alignment from human genomic DNA sequence information in 2001 and located on the q-arm of chromosome 14 (14q11.2) with two exons and encodes 161 amino acids (3). *Il-25* possesses approximately 16% to 20% homology with other IL-17 family members, and binds to specific homologous IL-17 receptors to transmit signals (4). Murine *Il-25* is located on chromosome 7 and encodes 160 amino acids, which has 80% sequence homology with the human *Il-25* (5).

Since the finding of IL-25, the exploration of the cellular origin of IL-25 has been dedicated. Initially, Fort et al. reported that IL-25 is mainly produced by T helper 2 (Th2) cells (4); subsequently, IKeda et al. found that mast cells may produce IL-25 by enhancing Th2-type immune response (6); and Kang et al. successfully used TiO_2_ to induce the secretion of IL-25 in alveolar macrophages *in vitro* (7); further studies identified tuft cells in the intestinal system also contributed to the expression of IL-25 (8, 9); Wang et al. found that human chorionic gonadotropin promoted the expression of IL-25 in decidual stromal cells (10); furthermore, IL-25 is also produced by activated eosinophils, basophils, liver cells, kidney cells, lung cells, innate immune cells, fibroblasts, and endothelial cells (11). In light of the above, IL-25 is widely secreted and expressed in various tissues and systems.

In fact, IL-25 has a dual role in regulating immune responses in different diseases. On the one hand, IL-25 is a driver of multiple allergic diseases (12). IL-25 is an amplifier of Th2 immune responses and binds to its receptor composed of interleukin 17 receptor A (IL-17RA) and IL-17RB for signal transduction (13). IL-25 activates nuclear factor of activated T cells c1 (NFATc1) and JunB transcription factors to induce the expression of interleukin 4 (IL-4), which enhances Th2 cell differentiation (2, 14, 15). Upon meeting with the stimulation of IL-25, nuclear transcription factor kappa B activator 1 (Act 1) binds to IL-17RB and then mediates the secretion of IL-5 and IL-13, which participates in the development of allergic diseases, *via* recruiting and activating eosinophils and stimulating the production of immunoglobulin E (IgE) (16–20). On the other hand, IL-25 also has positive effects. One of the characteristics of inflammatory bowel disease (IBD) is a low level of IL-25, and IL-25 treatment inhibits Toll-like receptors (TLRs)-induced inflammation and further alleviates the symptoms of IBD (21, 22). IL-25 suppresses IL-22-induced osteoclastogenesis *via* activation of signal transducer and activator of transcription 3 (STAT3) and p38 mitogen-Activated Protein Kinase (MAPK) pathway, participating in arthritic anti-inflammatory responses (19). Sonobe et al. reported that IL-25 down regulated the expression of junction adhesion molecule claudin 5 to maintain blood-brain barrier (BBB) in multiple sclerosis (23). Furthermore, IL-25 may also have anti-inflammatory effects in parasites infection, type 1 diabetes and systemic lupus erythematosus (2, 24).

IL-25 can be induced by a variety of factors, and the most common agents are allergens (25). IL-25 is highly expressed in the murine model of asthma established by ovalbumin (OVA) or house dust mite (26, 27). Pollen-allergic patients or pollen-challenged mice have an enhanced expression of IL-25 in the respiratory tract (28, 29). IL-25 can also be induced by environmental pollutants such as detergents, tobacco, ozone, particulate matter, diesel exhaust, nanoparticles and microplastic in the air or water, which damage epithelial cells and induce the secretion of IL-25 (30). What is of great interest is that pathogenic infection is also reported to be one of the factors that induce IL-25 secretion. It has reported that IL-25 is up-regulated during some parasitic, viral, and bacterial infections (31–33). Notably, the biofunction of IL-25 in infectious diseases is still unclear. Thus, in this review, we aim to briefly summarize the recent research progress on the functions of IL-25 in infectious diseases and probe into the potential therapeutic effects.

## The roles of IL-25 in parasitic infections

### Helminth infection

Helminths are multicellular organisms, and most of them are parasites including digenean flukes (trematodes), tapeworms (cestodes), and Nematoda (roundworms), which account for millions of infections worldwide (34). Helminths could disturb the host immune system, and the studies on helminths-host interactions focus on the fields of allergy and autoimmunity (35). Immunologically, chronic helminth infections are characterized by a skewing towards Th2 response, in which IL-25 plays a critical role (36, 37). IL-25 was strongly induced after multiple helminths infection in intestinal epithelial cells, including *Heligmosoides polygyrus* (*H. polygyrus*), *Trichuris muris* (*T. muris*), *Nippostrongylus brasiliensis* (*N. brasiliensis*), *Ascaris lumbricoides* (*A. lumbricoides)*, and *Schistosoma chinensis* (*S. chinensis*) (37, 38). As one of the main sources of IL-25 in human intestinal, tuft cells are rapidly increased during helminth infection (39). It is widely thought that IL-25 is an ‘alarmin’ for the helminth infection (40). Therefore, the biological function of IL-25 in the helminth infection is intriguing.

**Figure 1:**
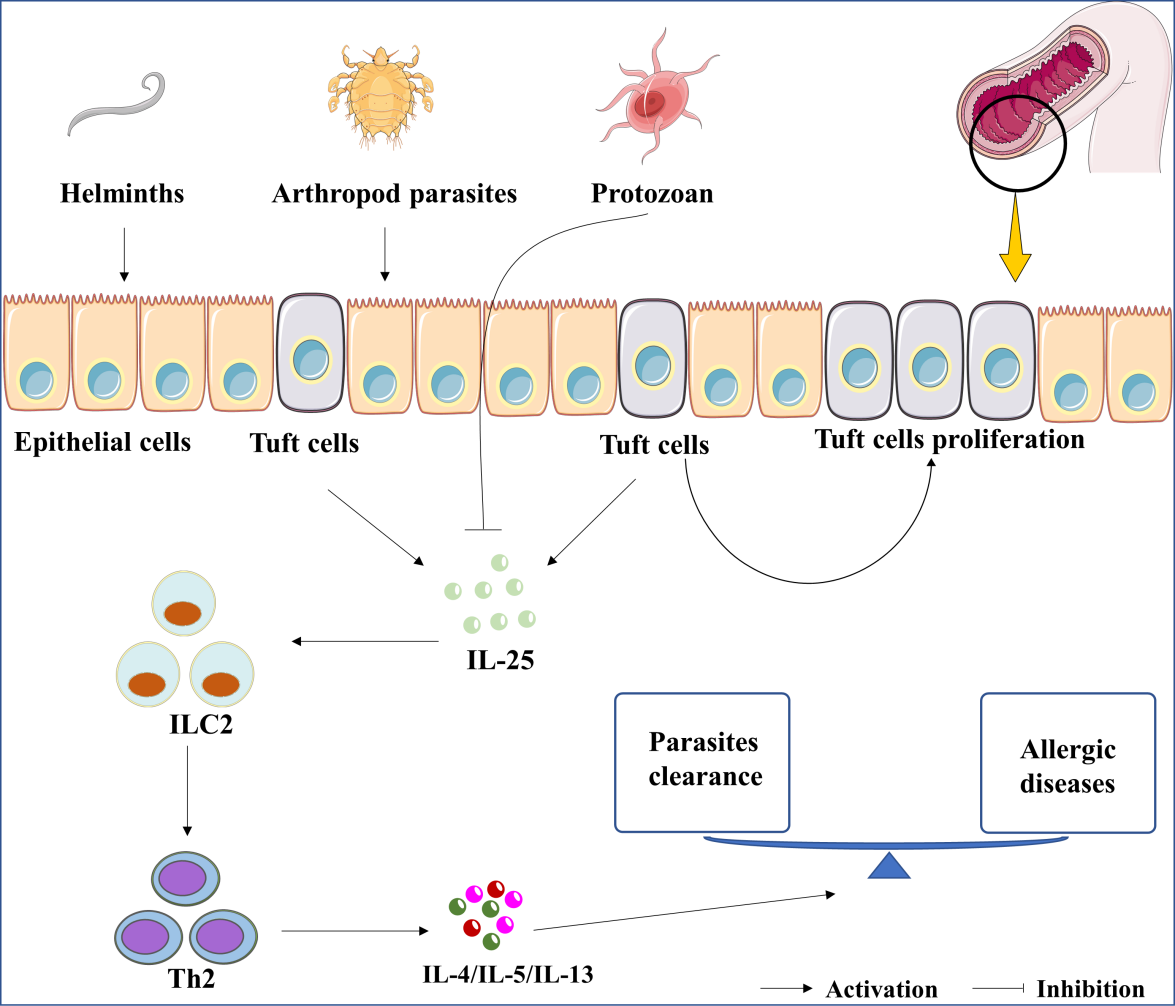
The roles of IL-25 in parasitic infection. The up-regulation of IL-25 was induced by the infection of parasites, including helminths and arthropod parasites. In contrast, IL-25 was downregulated by protozoan infections. Whatever, parasites-induced IL-25 or rIL-25 promotes the proliferation of tuft cells. Subsequently, the cytokines of IL-4, IL-5, and IL-13 are induced by IL-25/ILC2/Th2 axis to clean the parasite. Lastly, the expression of IL-4, IL-5, and IL-13 are associated with the development of asthma, allergic rhinitis, and atopic dermatitis.

First of all, IL-25 is crucial for the clearance of helminth infections. Previous studies showed that *Il-25* deficient mice exhibited an increased burden of helminth infection (41). *Il-17rb* deficient mice could not expel helminths in case of IL-25 overexpression (42). Group 2 innate lymphoid cells (ILC2) is considered as the innate counterpart of Th2 cells and participates in the host response against helminths (43). Fallon et al. clarified the protective role of ILC2 in preventing helminth colonization (44). Recombinant IL-25 (rIL-25) protein treatment promoted the expansion of ILC2s in helminths-infected-mice (45). IL-25-mediated-ILC2 activation resulted in the cooperation of Th2 and ILC2 and further promoted the secretion of Th2 cytokines, such as IL-4, IL-5, and IL-13 (36). These cytokines are involved in the recruitment of mast cells and eosinophil, which are important for the clearance of helminths (46). In short, the IL-25/ILC2/Th2 axis may be a potential therapeutic target for helminth infections, as shown in **Figure 1**. We perceive that the IL-25/ILC2/Th2 axis is responsible for the positive effect of the Th2 response in helminth infection related diseases.

Secondly, IL-25 also suppresses the inflammation caused by helminth infection. It is reported that *Il-25* deficient mice developed an exaggerated intestinal inflammation *via* increasing interferon gamma (IFN-γ) expression following *T. muris* infection (47). Overexpression of IL-25 could reduce IFNs expression (48). The cytokines of Th1 response, such as IL-1, IL-6, and tumor necrosis factor α (TNF-α), can be induced by the most of helminth infections (49–52), however, they can be reduced by IL-25 (53, 54). Kleinschek et al. discovered an elevated expression of TNF-α in *Il-25* deficient mice, and the administration of rIL-25 suppressed IL-1β and IL-6 expression in dendritic cells in mice (55), whose suppression will expand the response of Th2 cells.

Finally, IL-25 may also be involved in the pathogenic process of helminths. For instance, *Toxocara spp* larvae migrate to the lung and causing asthma-like symptoms, while IL-25 expression is one of the key cytokines for the development of asthma (56, 57). Vannella et al. reported that IL-25 induced by helminth infection was associated with the progression of pulmonary fibrosis (58). The development of cancer was also associated with helminth infections (59, 60). IL-25-activated ILC2s created an innate cancer-permissive microenvironment (61). However, there has been no direct evidence to verify that helminth infection-induced IL-25 is associated with tumorigenesis, which is a promising area that deserves us to explore in depth in the future.

### Protozoan infection

Protozoan parasites are single-celled organisms. Diarrheal diseases caused by intestinal protozoan parasites is a major food-borne public health problem worldwide (62). The infection induced by a protozoan parasite can reduce IL-25 expression while the administration of rIL-25 protein can help control amebiasis in mice (63). Similar to the helminth infection, IL-25 recruits ILC2, which mediates the clearance of protozoan parasites (64). In addition, IL-25 was downregulated in mice infected with *Plasmodium berghei* (*P. berghei*), and the incidence of parasitemia in the *Il-25* depleted mice were higher than that in the wild-type mice (65). Unfortunately, the underlying mechanism is unknown and this study ignored the therapeutic effect of rIL-25 on *P.berghei*. Given the previous evidence that IL-25 is extensively downregulated in the infection of protozoans and the supplement of IL-25 is beneficial for treating protozoan infections, more studies are needed to better elucidate the mechanism of IL-25 in protozoan infections.

### Arthropod parasites

Arthropod parasites are usually ectoparasitic and commonly include dust mites, ticks, and fleas (66). It is well known that house dust mite (HDMs) is a major factor for allergic diseases, such as atopic dermatitis, allergic rhinitis, and asthma (67). HDM-specific immunoglobulins in serum are positively correlated with IL-25 expression, and IL-25 involved in the development of atopic dermatitis, allergic rhinitis, and asthma sensitized to HDM (68). For atopic dermatitis, intelectin (ITLN) is a key factor for the expression of IL-25 in airway epithelial cells and aggravates allergic airway inflammation (69). The expression of IL-25 is up-regulated in nasal tissues from patients with allergic rhinitis (AR) (70); stimulation of Th2 cells with IL-25 locally promotes IL-13 and IL-9 production, which contributes to the pathology of allergic asthma (71). So far, the roles of IL-25 contributing to the pathogenesis of arthropod parasites infection are not fully understood, and increased attention is requisite.

## The roles of IL-25 in viral infections

### Respiratory syncytial virus (RSV)

RSV, as a contagious virus, can bring about acute respiratory tract infections, which is the primary cause of infant hospitalization worldwide (72). Although RSV infection usually manifests as a mild illness in healthy adults, it causes severe illness in the elderly or immunocompromised patients (73). RSV may infect the lower respiratory tract, resulting in an elevated risk of developing asthma (74). A previous study reported that *Il-17rb* deficient mice were protected from asthma aggravation deriving from RSV infection (75), establishing that the IL-17 family plays an important role in the pathogenesis of RSV-induced asthma. Meantime, after the RSV challenge, administration of anti-IL-25 antibody in mice prevents some pivotal features of asthma (76). It has been clarified that IL-25 acts on the progression of asthma and has been considered as a biomarker for the prognosis (77). Nevertheless, the way how IL-25 serves as RSV infection therapeutic remains unknown.

### Hepatitis C virus (HCV)

The infection of hepatitis viruses causes viral hepatitis. Five liver-specific viruses (A to E) exist, all of which have their own unique epidemiology, risk of liver complications, and responsiveness to antiviral therapies (78). HCV infection is one of the major causes of severe liver diseases such as chronic hepatitis, liver cirrhosis, and hepatocellular carcinoma (79). According to the World Health Organization (WHO), about 71 million people are infected with HCV worldwide, with at least 400,000 death annually (80). IL-17RA is expressed nearly in all types of liver cells (81). IL-25 was up-regulated in the serum of patients with HCV infection, and the levels of IL-25 in the serum were mainly associated with higher aspartate transaminase (AST) and alanine transaminase (ALT) (82). However, Cabral et al. reported that IL-25 could not be induced by the stimulation of HCV antigens in peripheral blood mononuclear cells (83), implying that IL-25 is not directly induced by HCV infection. IL-25 is also reported to be involved in the oncogenic effects of HCV (84). Tumor-associated macrophages (TAM), especially type 2 macrophages (M2), and their related cytokines are closely linked with the progression of hepatic cancer (85–87). Previous studies reported that IL-25 could induce M2 macrophage polarization, which was associated with the progression of hepatic cell carcinoma (HCC) (88, 89). However, the expression level of IL-25 in liver tissues with HCV infection is still not explored in the present decades. In addition, the mechanism of IL-25 in inflammation and chronic liver diseases has not been extensively studied. Thus, more studies are needed to better elucidate the effects of IL-25 in the development of viral hepatitis.

### Herpes simplex virus-1 (HSV-1)

HSV-1 is one of the most well-known members of the herpesviruses family, and about 70% of the population are infected with HSV-1 all over the world (90). About 3% of the patients with atopic dermatitis (AD), the most common chronic inflammatory skin disease in the world, are infected with HSV-1 and got eczema herpeticum (EH) (91). In addition to symptoms such as itching and soreness, HSV-1 infection poses a potentially life-threatening risk for AD patients (92). Previous study reported that the concentration of IL-25 in serum of AD patients was higher than control group (93), and IL-25 was involved in the pathogenesis of AD (94). IL-25 promoted the infection of HSV-1 among AD patients by suppressing the expression of interferon-gamma (IFN-γ), a key factor for the host to defend against viral infection (92, 95, 96). Subsequently, IL-25 treatment in the cell culture medium enhanced the replication of HSV-1 in keratinocytes (95). Ultimately, Salimi et al. confirm that IL-25-induced-ILC2s were present in mouse skin and exacerbated AD-like inflammation (97). The above evidences suggest that IL-25 is a key factor driving the process of AD following HSV-1 infection.

Herpes simplex keratitis (HSK) is a disease of the cornea caused by HSV-1 infection, and mice lacking *Il-17ra* showed a decreased severity of the lesion during HSV-1 infection (98), as well as a negligible damage to the epithelial layer, little fibrosis, and decreased infiltration of CD4^+^ T cells (99). Notably, *Il-17ra* deficiency makes a mouse unable to respond to any IL-17 family members. Until now, there are still lacking studies on the relationship between the IL-17 family and HSK, and the effect of IL-25/IL-17RA on HSK is still unclear, more studies are needed to elucidate it.

### Other viruses

**Figure 2:**
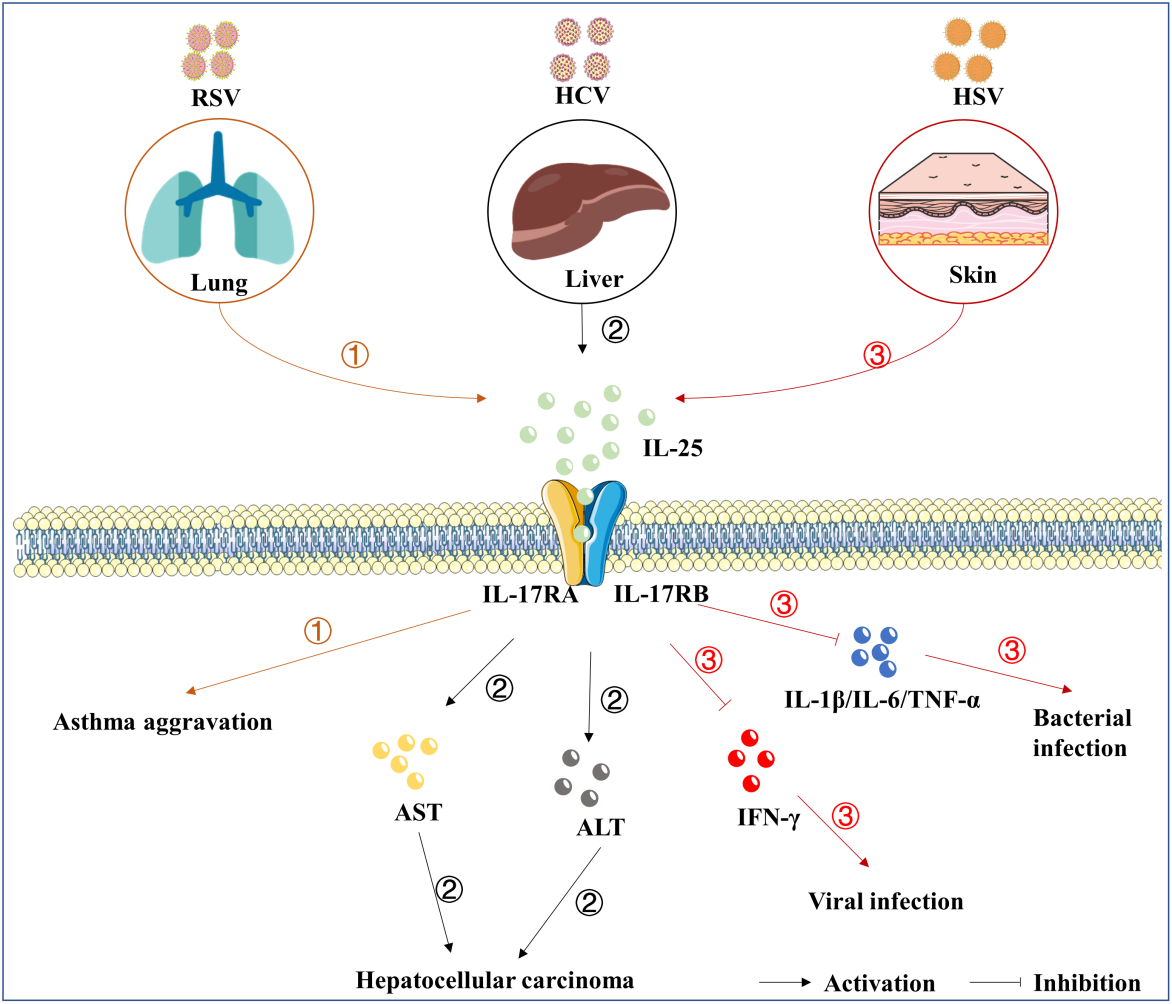
The roles of IL-25 in viral infection. IL-25 is up-regulated following the infection of RSV ①, HCV ②, and HSV-1 ③. Subsequently, IL-25 goes through the downstream signaling cascades by mediating the complex of receptors, consisting of IL-17RA and IL-17RB. IL-25 also induces the expression of IL-4/IL-5/IL-13, which are associated with the development of asthma. Furthermore, IL-25 overexpression was related to elevated AST and ALT involving chronic hepatitis, liver cirrhosis, and hepatocellular carcinoma. In addition, the up-regulation of IL-25 suppresses the cytokines of IFN-γ, IL-1β, IL-6, and TNF-α. However, direct evidence that IL-25 causes pathological damage is lacking.

IL-25 is also involved in the pathogenic process of multiple viruses. Beale et al. demonstrated that IL-25 was significantly induced in epithelial cells with rhinovirus (RHV) infection (100), and a higher level of IL-25 expression is associated with mucous metaplasia in RHV-infected infants and immature mice (101, 102). Haiyu et al. found that influenza virus (IAV) more powerfully induced the expression of IL-25 *in vitro*, and IL-25 positively correlated with the load of IAV (103). A respiratory allergic reaction caused by cytomegalovirus (CMV) is also associated with IL-25 secretion (104). IL-25 treatment exacerbates mouse intestinal West Nile virus (WNV) infection (105). A recent study showed that IL-25 blockade improved antiviral immunity during respiratory viral infection, and exogenous IL-25 treatment increased viral loads including rhinovirus and coronavirus (106). The evidence implies that IL-25 participates in the progression of many viral infectious diseases, as shown in **Figure 2**. However, the mechanisms of IL-25 associated with infectious diseases call for an urgent need to investigate.

## The roles of IL-25 in bacterial infections

Bacterial-derived metabolites or structural components are a strong activator of immune signaling pathways (107). IL-25 is one of the typical examples. The expression of IL-25 is regulated by pathogenic or commensal bacteria. *Francisella tularensis* (*Ft*) is a Gram-negative bacterium that causes tularemia (108). IL-25 strongly activates ILC2, which rapidly secretes IL-25 and results in a positive feed-back loop upon *Ft* infection, and the administration of IL-25 notably increases IgM production in mice; antibody-mediated depletion of ILC2 mainly supports the source of IL-5, which is required for IgM production; therefore, discovering the IL-25-ILC2-IL-5 axis is a novel strategy to improve vaccination depending on the IL-17RB signal pathway (109). *Clostridium difficile* (*C. difficile*) is a Gram-positive, spore-forming, anaerobic bacillus (110). *C. difficile* is widely distributed in the intestinal tract of humans and animals, and in the environment (111). Antibiotic-induced dysbiosis is the primary cause of the frequency and severity of *C. difficile* infection (CDI), which has become one of the most common hospital-acquired infections (112). Antibiotic-induced dysbiosis reduced colonic expression of IL-25, and fecal microbiota transplantation (FMT) could recover IL -25 expression by suppressing the expression of inflammatory genes (113). In addition, rIL-25 could also reduce the host mortality and the tissue pathology during the activating state of CDI infection (114). *Staphylococcus aureus* (*S. aureus*) strains infecting human brains may develop brain abscesses with the characteristics of inflammatory and septic lesions surrounded by fibrotic cysts (115). *S. aureus* can induce the release of IL-25 by epithelial cells (33). Compared with WT mice, *Il-17ra* deficient mice displayed a higher burdens of *S. aureus* in the brain; the influx of γ and δ T cells was increased in *Il-17ra* deficient mice following *S. aureus* infection; subsequently, the high infiltration of natural killer (NK) cells were absent in the brain abscesses in *Il-17ra* deficient mice, implying that IL-25 signaling played an important role in the regulation of adaptive immunity with the infection of *S. aureus* (116). However, IL-17A and IL-17F, which share IL-17RA with IL-25, play major roles in the host against bacterial infections (117). *Il-17ra* deficiency also makes a mouse unable to respond to IL-17A and IL-17F. Therefore, we should not ignore the effects of other members of the IL-17 family in the host to defend against bacterial infections.

**Figure 3:**
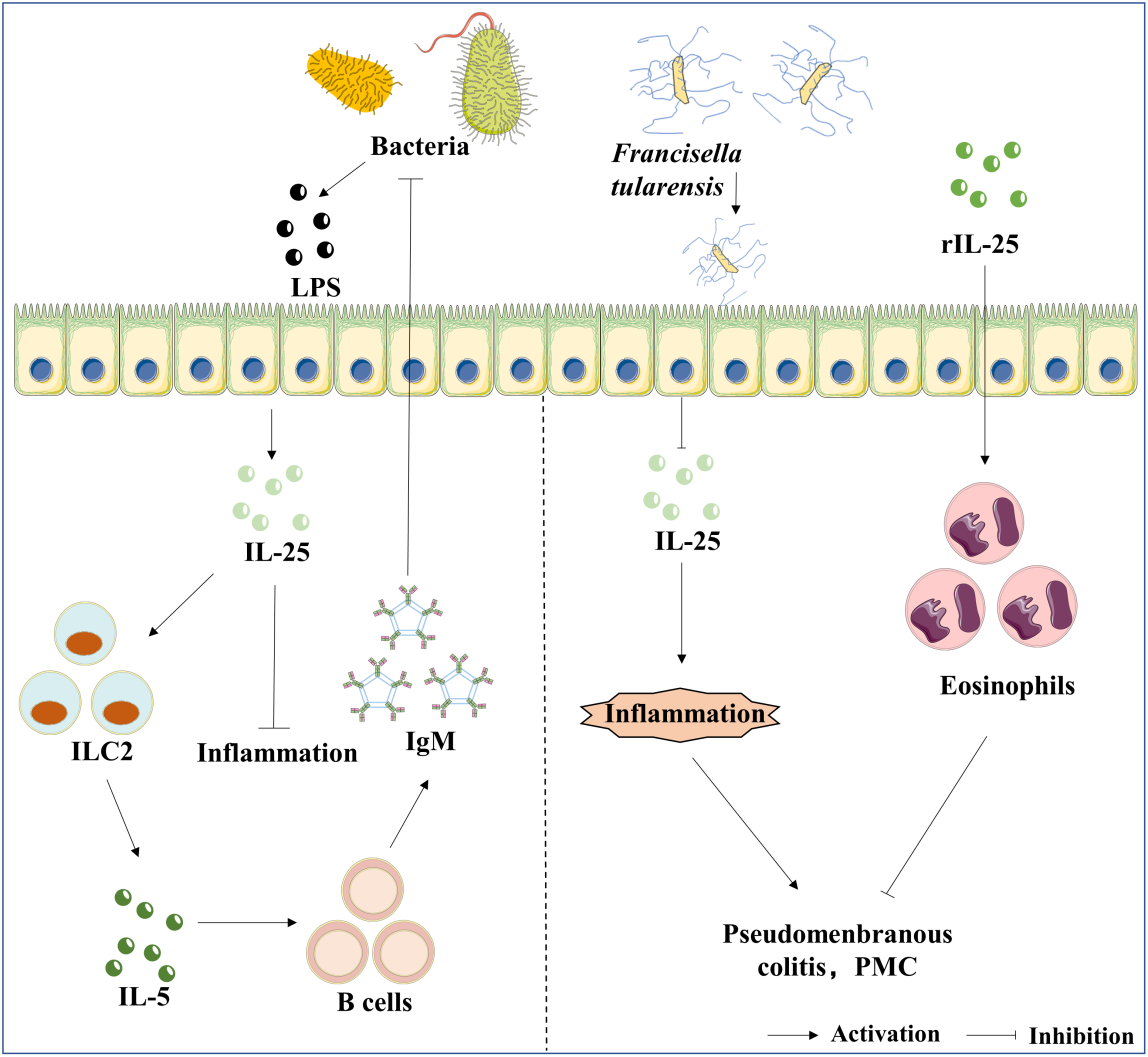
The roles of IL-25 in bacterial infection. LPS is the bacteria’s component, an inducer of IL-25 expression in epithelial cells. Furthermore, the axis of IL-25/ILC2/IL-5 mediates the production of IgM, and IgM exhibits a protective effect against bacterial infection. In contrast, *Ft* infection suppresses the expression of IL-25, which further results in pseudomembranous colitis (PMC). The administration of rIL-25 reduces the inflammatory storm caused by the infection of *Ft*.

Alterations in the composition of intestinal commensal bacteria are associated with enhanced susceptibility to multiple inflammatory diseases (118). The infection of intestinal commensal bacteria up-regulates the expression of IL-25 by intestinal epithelial cells and limits the expansion of Th17 cells in the intestine *via* inhibiting the expression of macrophage-derived IL-23, indicating that commensal bacteria influence intestinal immune homeostasis *via* direct regulation of the IL-25-IL-23-IL-17 axis (119). In summary, for bacterial infectious diseases, IL-25 is involved in the regulation of adaptive immunity and anti-inflammatory effect and influences the homeostasis of the intestinal immune through Th2 cells. In addition, IL-25 may have a potential therapeutic effect on treating bacterial infectious diseases, as shown in **Figure 3**, whereas the current studies are insufficient to give a definitive answer.

## Conclusions

IL-25 is a cytokine with a dual function in infectious diseases. In certain circumstances, IL-25 involves in the development of allergic diseases caused by the pathogenic infection. Most of pathogenic infection induces the up-regulation of IL-25. And then, IL-25 recruits ILC2 cells to promote the activation of Th2 cells. Th2 response up-regulates the expression of IL-4, IL-5, and IL-13, which are essential for allergic diseases caused by the pathogenic infection. Meanwhile, IL-25 plays an important role in the clearance of pathogens. Mechanically, IL-4, IL-5, and IL-13-induced by the expression of IL-25 to enhance the differentiation of Th2 cell and stimulate the production of IgE and IgM by B cells, as well as recruit and activate eosinophils, mast cells and basophils to perform the function of the clearance of pathogens. Therefore, it’s feasible to use IL-25 as a novel therapeutic target for infectious diseases. However, IL-25 treatment inhibits the expression of IFN-γ, which is beneficial for the infection of HSV-1. Thus, further investigations are necessary to explore the different mechanisms induced by IL-25 expression in different infectious diseases. Remarkably, given the circumstance *Il-17ra* deficiency also makes a mouse unable to respond to many IL-17 family members, any interpretation of IL-25 based on *Il-17ra* deficiency in this review should be made with caution in the future.

## Author contributions

DC and JW contributed to the conception and design of the current review article. JW was responsible for drafting the manuscript. JW and FZ revised the manuscript. FZ, HT, and WN helped to draw the figures in the paper. ZW and DC performed the manuscript review and writing-review and editing. ZW provided the funding to support the review. All authors contributed to the article and approved the submitted version.

## Funding

This work was supported by grants from the National Natural Science Foundation of China (Grant Number: 81900823 to DC and 31970149 to ZW).

## Conflict of interest

The authors declare that the research was conducted in the absence of any commercial or financial relationships that could be construed as a potential conflict of interest.

## Publisher’s note

All claims expressed in this article are solely those of the authors and do not necessarily represent those of their affiliated organizations, or those of the publisher, the editors and the reviewers. Any product that may be evaluated in this article, or claim that may be made by its manufacturer, is not guaranteed or endorsed by the publisher.
